# Ageing alters cysteine oxidation‐regulated redox signalling in skeletal muscle: Integrative omics and AI‐based structural predictions

**DOI:** 10.1113/EP093750

**Published:** 2026-07-11

**Authors:** Ufuk Ersoy, Malcolm J. Jackson

**Affiliations:** ^1^ Department of Musculoskeletal and Ageing Sciences, Institute of Life Course and Medical Sciences University of Liverpool Liverpool UK

**Keywords:** ageing, alphafold3, cysteine, proteomics, reactive oxygen species, skeletal muscle

## Abstract

Ageing is associated with loss of skeletal muscle mass and strength (sarcopenia) and disrupted redox homeostasis. Redox signalling is essential for muscle adaptation, yet the mechanisms by which ageing disrupts cysteine‐based regulation are poorly defined. The drivers of site‐specific reactivity and signalling specificity in aged muscle remain unknown. Here, we interrogated the *OxiMouse* dataset to map age‐related cysteine oxidation in skeletal muscle and, using AI, simulate oxidative modifications at key cysteine residues to predict structural and functional consequences for specific proteins. Ageing was found to remodel the redox landscape through selective oxidation of discrete cysteine residues, in a site‐specific manner, even within the same protein. These findings support that ageing drives pathway‐targeted modulation of protein function rather than a uniform, global oxidative shift. Moreover, age‐related cysteine oxidation is not randomly distributed but appears to target interconnected protein networks involved in mitochondrial metabolic pathways, muscle function and proteostasis, indicating a coordinated remodelling in redox signalling as a hallmark of skeletal muscle ageing. To connect proteomic signatures to mechanisms, AlphaFold3 was used to simulate progressive cysteine oxidation and predict structural outcomes. Protein docking simulations were then performed using HADDOCK. This approach was applied to prioritise functionally important cysteines identified in the dataset. These results suggest that skeletal muscle ageing drives selective rewiring of physiologically relevant cysteine‐based redox signalling networks. By integrating redox proteomics with AI‐based structural simulation, this study provides a framework to prioritise key oxidation‐sensitive cysteines, including within the 26S proteasome, as potential mechanistic nodes and intervention targets for sarcopenia.

## Abstract (highlights)

- **What is the central question of this study?**
  This study investigates how ageing reshapes cysteine oxidation in skeletal muscle, whether these alterations reflect a global oxidative shift or selective, site‐specific remodelling, and which oxidised sites are most likely to have structural/functional consequences for specific proteins.
- **What is the main finding and its importance?**
  Ageing is associated with selective, network‐enriched cysteine oxidation in proteins regulating mitochondrial metabolism, muscle contraction and proteostasis, indicating a coordinated remodelling in redox signalling as a hallmark of skeletal muscle ageing. Integrating redox proteomics with AI‐based structural modelling has also been found to help prioritise oxidation‐sensitive cysteines as mechanistic nodes and potential intervention targets for sarcopenia.

## 1 INTRODUCTION

Over the past two centuries, average human life expectancy has increased markedly, whereas disease‐free lifespan (health span) has not kept pace (Oeppen & Vaupel, 2002). As a result, most individuals now live far beyond the ages shaped by evolutionary selection, leading to a rising incidence of chronic age‐related diseases (Partridge et al., 2018). Among these is sarcopenia, the progressive loss of skeletal muscle mass and strength that profoundly impacts mobility, metabolic health and quality of life in older adults (Cruz‐Jentoft et al., 2019).

Ageing is accompanied by widespread alterations in cellular physiology and a progressive decline in the capacity to maintain systemic homeostasis. These changes arise from a series of mechanistic hallmarks, whose relative contributions differ across different tissues and cell types (López‐Otín et al., 2023). In the 1950s, Denham Harman first proposed the free radical theory of ageing, suggesting that reactive oxygen species (ROS) generated during normal metabolism drive ageing through cumulative oxidative damage (Harman, 1955). Elevated ROS levels can cause irreversible oxidation of proteins, DNA and lipids, ultimately contributing to cellular dysfunction, death and disease. More recently, however, ROS have emerged as critical regulators of cellular function. At physiological levels, they act as signalling molecules that reversibly modify target proteins, thereby regulating their activity, localisation and interactions (D'Autréaux & Toledano, 2007; Lennicke & Cochemé, 2021; Paulsen & Carroll, 2013). This redox signalling influences diverse processes, including transcription factor activation, gene expression, epigenetic regulation and metabolic enzyme activity (Schieber & Chandel, 2014).

Cellular ROS levels are tightly regulated through mechanisms that control their generation and their removal via antioxidant systems and cofactors (Phaniendra et al., 2015). In skeletal muscle, major sources of superoxide (O_2_•^−^)/hydrogen peroxide (H_2_O_2_) include mitochondrial electron transport chain (ETC) complexes I and III and NADPH oxidases (NOXs). H_2_O_2_ is membrane‐permeant and traverses membranes via facilitated transport through aquaporins, positioning it as a critical regulator of redox signalling (Lambeth, 2004; Murphy, 2009; Sies & Jones, 2020).

Longer‐term metabolic adaptations are accomplished through changes in gene and protein expression. In contrast, short‐term metabolic processes are controlled by allosteric effectors and/or post‐translational modifications, including oxidative modifications (Dickinson & Chang, 2011; Walsh et al., 2005). Many ROS‐dependent signalling events are mediated by reversible oxidation of thiol groups on cysteine residues in target proteins, allowing cysteine oxidation to function as a redox switch in protein regulation (Lennicke & Cochemé, 2021). Although cysteine residues are among the least abundant amino acids, approximately 90% of cysteine residues are evolutionarily conserved, reflecting their functional importance in redox regulation (Cremers & Jakob, 2013; Fomenko et al., 2008). However, not all cysteines are equally reactive. Their susceptibility to oxidation is strongly influenced by the protein's structural context. Surface‐exposed cysteines are more likely to be redox‐active, participating in catalytic and regulatory processes, whereas buried cysteines often contribute to protein stability through forming intramolecular disulfide bonds or coordination of prosthetic groups such as iron–sulfur (FeS) clusters (Go et al., 2015; Lennicke & Cochemé, 2021). Many intracellular pathways, including insulin signalling, mitochondrial function/homeostasis and energy metabolism, circadian rhythm and proteostasis, respond dynamically to changes in the cellular redox balance (Lennicke & Cochemé, 2021; Mitsuishi et al., 2012; Reinke & Asher, 2019; Topf et al., 2018; Yin et al., 2021).

Despite its clinical importance, the molecular drivers of sarcopenia remain incompletely understood, limiting the development of effective interventions. Emerging evidence implicates redox dysregulation and aberrant ROS signalling as key contributors to age‐related declines in muscle mass and function (Dos Santos et al., 2015; Jackson, 2025; Li et al., 2015; Short et al., 2005; Vasilaki et al., 2006). Evidence‐based clinical practice guidelines advocate resistance exercise as one of the primary strategies for treating sarcopenia (Sayer & Cruz‐Jentoft, 2022). Contractile activity during resistance exercise increases ROS production, which plays essential roles in muscle physiology and adaptation. In healthy muscle, transient elevations in ROS during exercise act as crucial signalling events, stimulating mitochondrial biogenesis, enhancing antioxidant defences and promoting hypertrophic adaptations (Henriquez‐Olguin et al., 2019, 2020; McArdle et al., 2019; Powers & Jackson, 2008). These effects are largely mediated by specific oxidative modifications of cysteine residues in redox‐sensitive proteins (Sobotta et al., 2015; Winterbourn & Hampton, 2015). Ageing disrupts this finely tuned redox signalling. Aged skeletal muscle is characterised by chronically elevated ROS levels and a diminished capacity to activate redox‐sensitive pathways (Derbré et al., 2012; McArdle et al., 2001; Palomero et al., 2013). These impaired redox responses likely contribute to sarcopenia by attenuating key redox‐regulated signalling cascades, compromising mitochondrial biogenesis and reducing the efficacy of hypertrophic stimuli (Cuthbertson et al., 2005; Vasilaki et al., 2006; Viña et al., 2009).

Although redox signalling is central to skeletal muscle adaptation, the mechanisms by which ageing disrupts redox‐dependent regulation remain poorly defined. While many redox‐sensitive cysteine residues have been identified, the determinants of signalling specificity and reactivity in aged skeletal muscle are not well understood. Here, we interrogated the *OxiMouse* dataset to map age‐related cysteine oxidation in skeletal muscle and, using AI, simulate oxidative modifications at key residues to predict structural and functional consequences. This integrative analysis uncovers previously unappreciated patterns of redox dysregulation during ageing and suggests a mechanistic link to age‐related muscle loss.

## 2 METHODS

### 2.1 Data source

The quantitative dataset of stoichiometric cysteine oxidation in young (16‐week) and old (80‐week) C57BL/6J mouse skeletal muscle was downloaded from https://oximouse.hms.harvard.edu/ (Xiao et al., 2020). This dataset contains the average percentage of reversible cysteine oxidation (sulfenic acid state) for five biological replicates at each age.

### 2.2 Cysteine oxidation site‐level statistical analysis and data analysis

Statistical significance was assessed with a Welch‐style pseudo *t*‐test derived from group means, standard errors and sample sizes (*n* = 5 per group). The reported standard errors were used to estimate standard deviations, and degrees of freedom were calculated with the Welch–Satterthwaite approximation. Resulting *P*‐values were adjusted for multiple testing using the Benjamini–Hochberg false discovery rate (FDR) method. Sites were classified as significantly regulated if they met both an absolute difference in oxidation ≥10% and an adjusted *P*‐value threshold of FDR < 0.05 (Xiao et al., 2020).

All analyses were conducted in R (version 4.4.1). Volcano plots were generated using ggplot2 (version 4.0.1) to visualise differences in cysteine oxidation between samples from skeletal muscles of young and old mice. Gene Ontology (GO) annotations for mouse proteins were retrieved using the org.Mm.eg.db package (version 3.20.0), and cysteine residues identified in the *OxiMouse* dataset were mapped to genes associated with specific biological processes, including mitochondrion, proteostasis, inflammatory signalling, nucleic acid metabolism, gene expression, and cellular stress responses. GO terms and their inferred annotations (GOALL key type) were used to group proteins for functional interpretation.

Local sequence motifs surrounding oxidised cysteine residues were extracted from *OxiMouse* data by identifying the modified cysteine (C#) and retrieving a ±4 residue window. For each motif, the presence of individual amino acids was assessed after removal of the central cysteine. Amino acid locality enrichment was then calculated as the percentage‐point difference between foreground (highly oxidised sites, >20% oxidation) and background probabilities $\left(P_{\mathrm{foreground}}-P_{\mathrm{background}}\right)$. Position weight matrices (PWMs) were computed separately for foreground and background motifs, and enrichment was visualised as log_2_ foreground/background ratios using sequence logos generated using ggseqlogo (version 0.2.2). Enrichment analyses were conducted separately for the young and old samples.

The BioPlex 3.0 protein–protein interaction (PPI) network was used and proteins containing significantly differentially oxidised cysteine sites were mapped onto this network (Huttlin et al., 2021). Network visualisation was performed in R. Because BioPlex is a human interactome, mouse proteins were converted to their human orthologues before network mapping. Orthology mapping was performed using Ensembl BioMart (biomaRt, version 2.62.1), retaining one‐to‐one orthologues where available; for one‐to‐many mappings, the orthologue with the highest confidence/annotation support was selected.

KEGG pathway enrichment analysis was conducted using the clusterProfiler package (version 4.14.6). UniProt accessions were mapped to human Entrez Gene IDs and enrichment was performed with enrichKEGG using the human KEGG organism code (‘hsa’). Enriched pathways were converted to gene symbols for readability, and fold enrichment was calculated from gene and background ratios. Targets identified through KEGG enrichment were subsequently mapped onto the BioPlex 3.0 network to identify coordinated oxidation events within biological pathways.

### 2.3 Molecular dynamics simulations

AlphaFold3 (version 3.0.1‐96‐g2e3703e) was used for in silico structural modelling of wild‐type (WT) and mutant/modified proteins, using default inference settings (Abramson et al., 2024). Protein docking simulations were performed using HADDOCK 2.4, following standard procedures. Final clusters were ranked by HADDOCK score (a weighted sum of Evdw, Eelec, Edesolv and other terms), cluster size, root mean square deviation (RMSD) from the lowest energy structure, buried surface area, restraint violation energy and *Z*‐score as described in the HADDOCK protocol literature. The top‐ranked cluster was selected for further analysis (Honorato et al., 2021, 2024).

## 3 RESULTS

### 3.1 Redox regulation is highly specialised and context‐dependent

**FIGURE 1:**
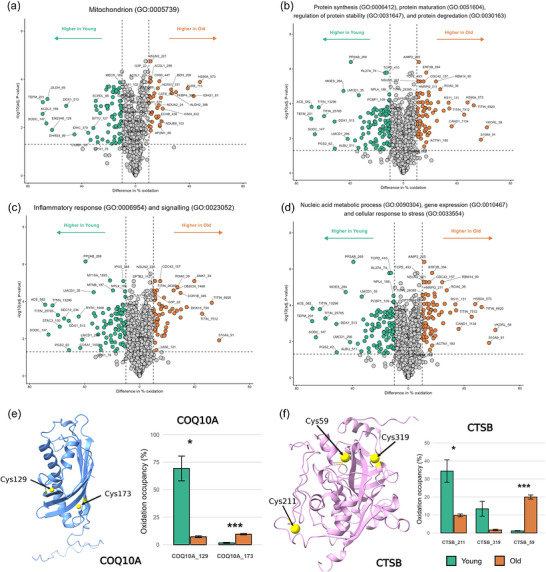
Systematic remodelling of redox signalling networks in ageing skeletal muscle reveals pathway‐specific patterns and site‐level selectivity. (a–d) Volcano plots showing differences in cysteine oxidation between old and young skeletal muscle mapped to Gene Ontology (GO) categories aligned with ageing hallmarks. Each point represents an oxidation site; the *x*‐axis shows the difference in oxidation, and the *y*‐axis indicates −log10(adjusted *P*‐value). Functional clusters include: (a) mitochondrion (GO:0005739), (b) proteostasis pathways encompassing protein synthesis, maturation, and degradation (GO:0006412, GO:0051604, GO:0030163), (c) inflammatory response and signalling (GO:0006954, GO:0023052), and (d) nucleic acid metabolism, gene expression, and stress‐responsive processes (GO:0090304, GO:0010467, GO:0033554). (e) COQ10A exhibits different oxidation patterns at Cys129 and Cys173 in young muscle. (f) CTSB shows differential oxidation at Cys59, Cys211 and Cys319 between age groups. Structures highlight oxidised cysteines (yellow), and bar plots below depict oxidation occupancy (% ±SE) for young (green) and old (orange) muscle. Statistical significance was assessed using pseudo *t*‐tests based on group means, SEs and sample sizes, followed by Benjamini–Hochberg FDR correction. Sites were considered significant if FDR < 0.05 and the absolute difference ≥10%. Data are presented as means ± SEM. Significance levels: *P < 0.05, ***P < 0.001.

The *OxiMouse* platform detected ∼5500 unique cysteine sites in skeletal muscle (Supporting information, Table S1), an ∼4‐fold increase in coverage over conventional redox workflows that typically quantify ∼1400 cysteine sites with limited stoichiometric resolution (McDonagh et al., 2015). Using this expanded atlas, we applied a site‐centric pseudo‐statistical approach to quantify age‐dependent thiol oxidation and then mapped the resulting site‐level changes to GO categories aligned with the established hallmarks of ageing. This analysis revealed heterogeneous, pathway‐specific redox patterns between young and old mice (Figure 1). Cysteine sites within proteins involved in mitochondrial function and homeostasis (GO:0005739; Figure 1a) exhibited distinct age‐related differences in oxidation. Similarly, pathways associated with proteostasis (protein translation, proteasome‐mediated protein catabolism, protein processing, and cellular responses to stress; GO:0006412, GO:0051604, GO:0030163, GO:0033554) displayed pronounced, bidirectional differences with ageing (Figure 1b). Age‐dependent redox alterations were also evident in proteins related to inflammatory responses and signalling (GO:0006954, GO:0023052; Figure 1c), as well as those involved in nucleic acid metabolism, gene expression, and stress‐responsive processes (GO:0090304, GO:0010467, GO:0033554; Figure 1d).

Notably, even cysteine residues within the same protein showed differential oxidation; for example, coenzyme Q10A (COQ10A), a key enzyme in the mitochondrial ETC and ATP production, displayed markedly different levels of cysteine oxidation at sites 129 and 173 (Figure 1e). Growing evidence, supported by clinical studies, demonstrates that cathepsins act as key regulators of muscle mass and represent novel opportunities for therapeutic intervention (Hou et al., 2025; Moon et al., 2016; Pinto et al., 2025). Cathepsin B (CTSB), a lysosomal cysteine protease, demonstrated different oxidation patterns at sites 59, 211 and 319 between young and old mice (Figure 1f). Collectively, these findings support the concept that ageing reshapes the redox landscape through selective modification of discrete cysteine residues, even within the same protein, thereby enabling pathway‐specific modulation of protein function rather than global oxidative shifts.

**FIGURE 2:**
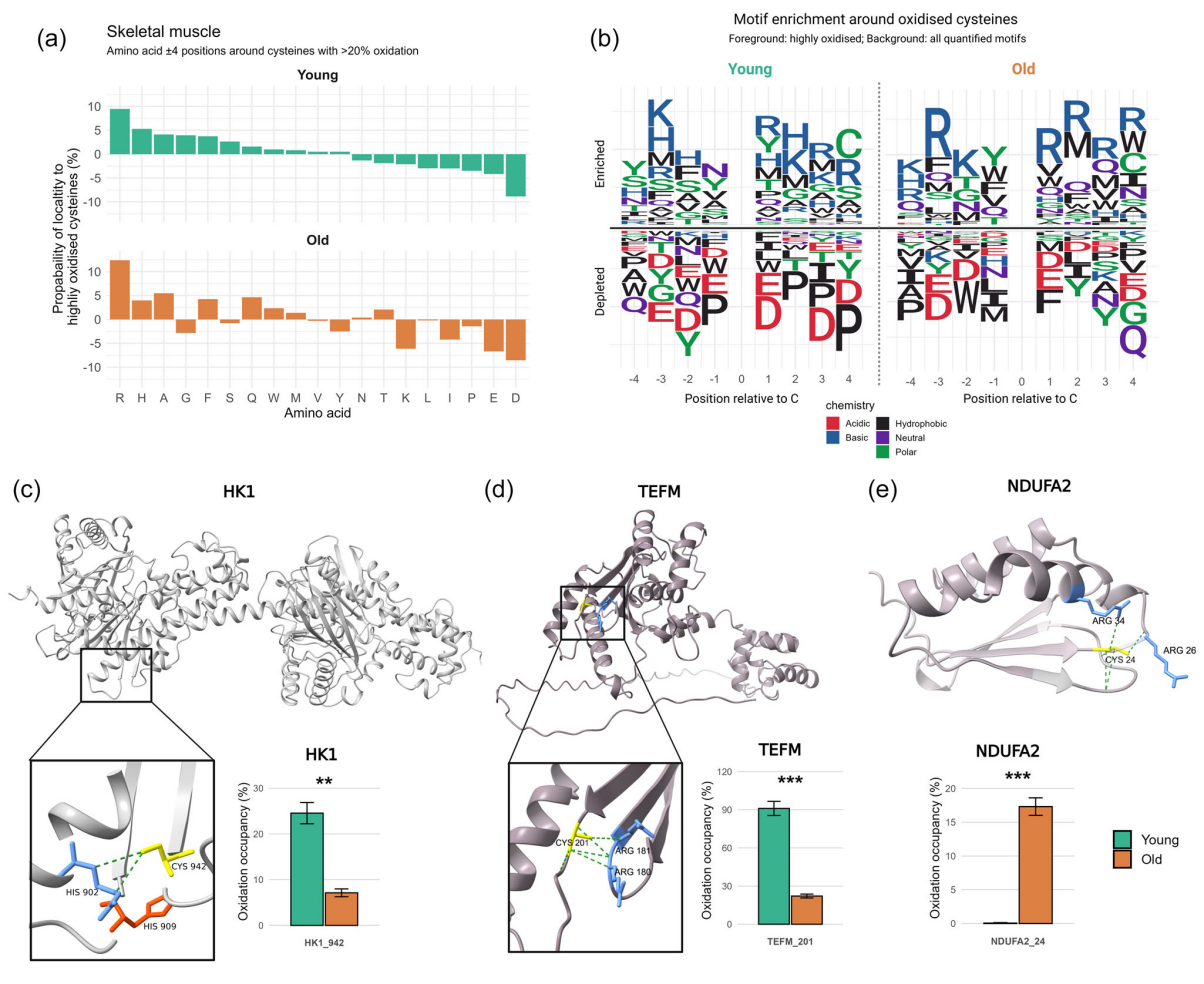
Proximal amino acid environment governs cysteine oxidation in skeletal muscle with modest age‐related effects. (a) Probability of amino acid occurrence ±4 positions around cysteines exhibiting >20% oxidation in young (top) and old (bottom) skeletal muscle. Positive values indicate enrichment; negative values indicate depletion relative to all cysteines quantified. (b) Motif enrichment plots demonstrating amino acid frequency surrounding oxidised cysteines in young (left) and old (right) muscle. Amino acids are coloured by chemical property: acidic (red), basic (blue), hydrophobic (black), neutral (purple) and polar (green). (c–e) Representative 3D structures of HK1 (c), TEFM (d), and NDUFA2 (e), highlighting oxidised cysteine residues (yellow) and proximal arginine residues (blue). Statistical significance was assessed using pseudo *t*‐tests based on group means, SEs and sample sizes, followed by Benjamini–Hochberg FDR correction. Sites were considered significant if FDR < 0.05 and the absolute difference ≥10%. Data are presented as mean ± SEM. Statistical significance: ***P < 0.001, ****P < 0.0001.

Although the data above suggest precise redox tuning rather than generalised oxidative drift with age, the mechanism behind this selectivity remains unclear. It was suggested that neighbouring residues tune the thiol microenvironment and thereby influence oxidative modification tendency (Xiao et al., 2020). In support of this, analysis of *OxiMouse* data revealed that the amino acid composition within ±4 positions of cysteines with >20% oxidation exhibits an enrichment of basic residues (notably arginine) and a depletion of acidic residues (aspartic acid and glutamic acid) in skeletal muscle, with age‐specific variations (Figure 2a). Furthermore, motif analysis revealed subtle differences in the sequence signatures surrounding oxidised cysteines in young versus old muscle (Figure 2b). However, cysteine redox sensitivity is fundamentally a three‐dimensional electrostatic property; linear motifs reflect a bias but do not fully determine oxidation state. Structural analysis illustrated this principle: in Hexokinase1 (HK1), one highly oxidised cysteine residue is adjacent to arginine and histidine residues in the 3D structure of the protein (Figure 2c). In transcription elongation factor, mitochondrial (TEFM), Cys201 exhibited ∼90% oxidation in muscle from young despite no proximal arginine in sequence; 3D modelling shows Arg180–181 contacting Cys201 (Figure 2d). Similarly, in NADH dehydrogenase [ubiquinone] 1 alpha subcomplex subunit 2 (NDUFA2), arginine residues positioned near the highly oxidised Cys24 site, underscoring the role of spatial electrostatics in redox selectivity (Figure 2e).

### 3.2 Age‐related cysteine oxidation reveals coordinated protein interaction networks and pathway remodelling in mouse skeletal muscle

**FIGURE 3:**
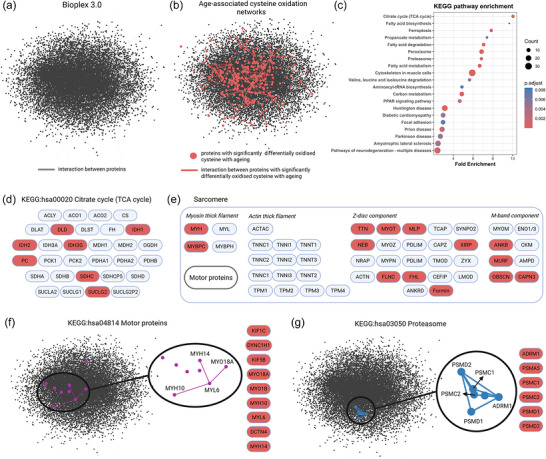
Age‐related cysteine oxidation reveals coordinated protein interaction networks and pathway remodelling in mouse skeletal muscle. (a) Bioplex 3.0 protein–protein interaction (PPI) network. (b) Proteins with significantly differentially oxidised cysteine sites with ageing (*P* < 0.05, |Δoxidation| > 10) mapped onto the BioPlex 3.0 network, revealing extensive interconnectivity. (c) KEGG pathway enrichment analysis of proteins with age‐related cysteine oxidation. Dot size denotes protein count per pathway; colour indicates adjusted *P*‐value, ranging from blue (lower significance) to red (higher significance). (d) Citrate cycle (TCA cycle) enzymes showing significant age‐related cysteine oxidation. (e) Sarcomere proteins across thick filaments, Z‐discs and M‐bands demonstrating age‐related cysteine oxidation. (f) Motor protein subnetwork containing significantly oxidised cysteine residues. (g) Proteasome subnetwork highlighting coordinated cysteine oxidation of core and regulatory subunits.

To investigate the systems‐level effect of ageing on protein cysteine oxidation in skeletal muscle, we integrated proteins containing significantly differentially oxidised cysteine sites (*P* < 0.05 and |Δoxidation| > 10, Supporting information, Table S2) with the BioPlex 3.0 PPI network (Huttlin et al., 2021) and performed KEGG pathway enrichment analysis (Figure 3) (Kanehisa & Goto, 2000). Mapping site‐specific redox differences onto a comprehensive interactome enables the identification of coordinated cysteine modifications across functionally related proteins, while KEGG analysis provides curated pathway‐level interpretation of these targets.

The global BioPlex 3.0 PPI network consists of dense and highly interconnected protein clusters (Figure 3a). Mapping the proteins containing significantly differentially oxidised cysteine residues in aged skeletal muscle onto this network revealed extensive interconnectivity among these proteins (Figure 3b and Supporting information, Table S3). This non‐random clustering suggests that age‐related cysteine oxidation follows a coordinated, network‐level pattern rather than occurring as isolated events, suggesting structured redox regulation during ageing.

KEGG pathway analysis identified strong over‐representation of metabolic and protein homeostasis‐associated pathways, including citrate cycle (TCA cycle), fatty acid metabolism (biosynthesis and degradation), peroxisome function, proteasome and ferroptosis. In addition, pathways related to muscle function and structure (e.g., cytoskeleton in muscle cells) and neurodegenerative diseases (e.g., amyotrophic lateral sclerosis) were significantly enriched, suggesting broad functional consequences of age‐related changes in redox signalling (Figure 3c and Supporting information, Table S4).

Within the KEGG:hsa00020 citrate cycle (TCA cycle), multiple core enzymes exhibited significant age‐related cysteine oxidation, including dihydrolipoamide dehydrogenase (DLD), isocitrate dehydrogenase isoforms (IDH1, IDH2 and IDH3G), pyruvate carboxylase (PC), succinate dehydrogenase subunit C (SDHC) and succinyl‐CoA ligase subunit G2 (SUCLG2) (Figure 3d) (Arnold & Finley, 2023). These proteins span several enzymatic steps of the TCA cycle, and their coordinated oxidation suggests widespread redox remodelling of mitochondrial energy metabolism during ageing.

Notably, extensive age‐related cysteine oxidation was observed across multiple structural and regulatory components of the sarcomere (Figure 3e). These included myosin heavy chains (MYHs), myosin‐binding protein C (MYBPC), titin (TTN), nebulin (NEB), filamin C (FLNC), LIM domain proteins such as four‐and‐a‐half LIM domains (FHL) and muscle LIM protein (MLP), as well as Z‐disc and M‐band constituents including myotilin (MYOT), telethonin (TCAP), obscurin (OBSCN) and calpain 3 (CAPN3). In addition to sarcomere proteins, several motor proteins, including myosin light chain 6 (MYL6), MYH isoforms MYH10 and MYH14 and unconventional myosin‐XVIIIa (MYO18A), displayed significant age‐related cysteine oxidation and formed a tightly connected subnetwork within the PPI landscape (Figure 3f). The widespread distribution of redox modifications across thick filaments, Z‐discs and M‐bands highlights a potential role for cysteine oxidation in regulating sarcomere integrity, force transmission and mechano‐transduction during skeletal muscle ageing.

Finally, a distinct oxidised subnetwork was identified within the proteasome complex (Figure 3g). Multiple core and regulatory proteasome subunits, including adhesion regulating molecule 1 (ADRM1), proteasome subunit alpha 5 (PSMA5), 26S proteasome ATPase subunits (PSMC1 and PSMC2), and 26S proteasome non‐ATPase regulatory subunits (PSMD1 and PSMD2), exhibited coordinated cysteine oxidation and strong interconnectivity (Tanaka, 2009). These findings suggest that ageing is associated with redox modulation of protein quality control and degradation pathways, potentially contributing to altered proteostasis in aged skeletal muscle.

Collectively, these results indicate that ageing‐related cysteine oxidation is not randomly distributed but instead targets interconnected protein networks involved in mitochondrial metabolic pathways, muscle structure and function, and proteostasis, pointing to coordinated remodelling in redox signalling as a hallmark of skeletal muscle ageing.

### 3.3 Integrating redox proteomics with AI‐based modelling can prioritise oxidation‐sensitive cysteines as key mechanistic and intervention targets for sarcopenia

#### 3.3.1 Age‐related remodelling of the SOD1 disulfide network is predicted to perturb the metal‐site microenvironment

**FIGURE 4:**
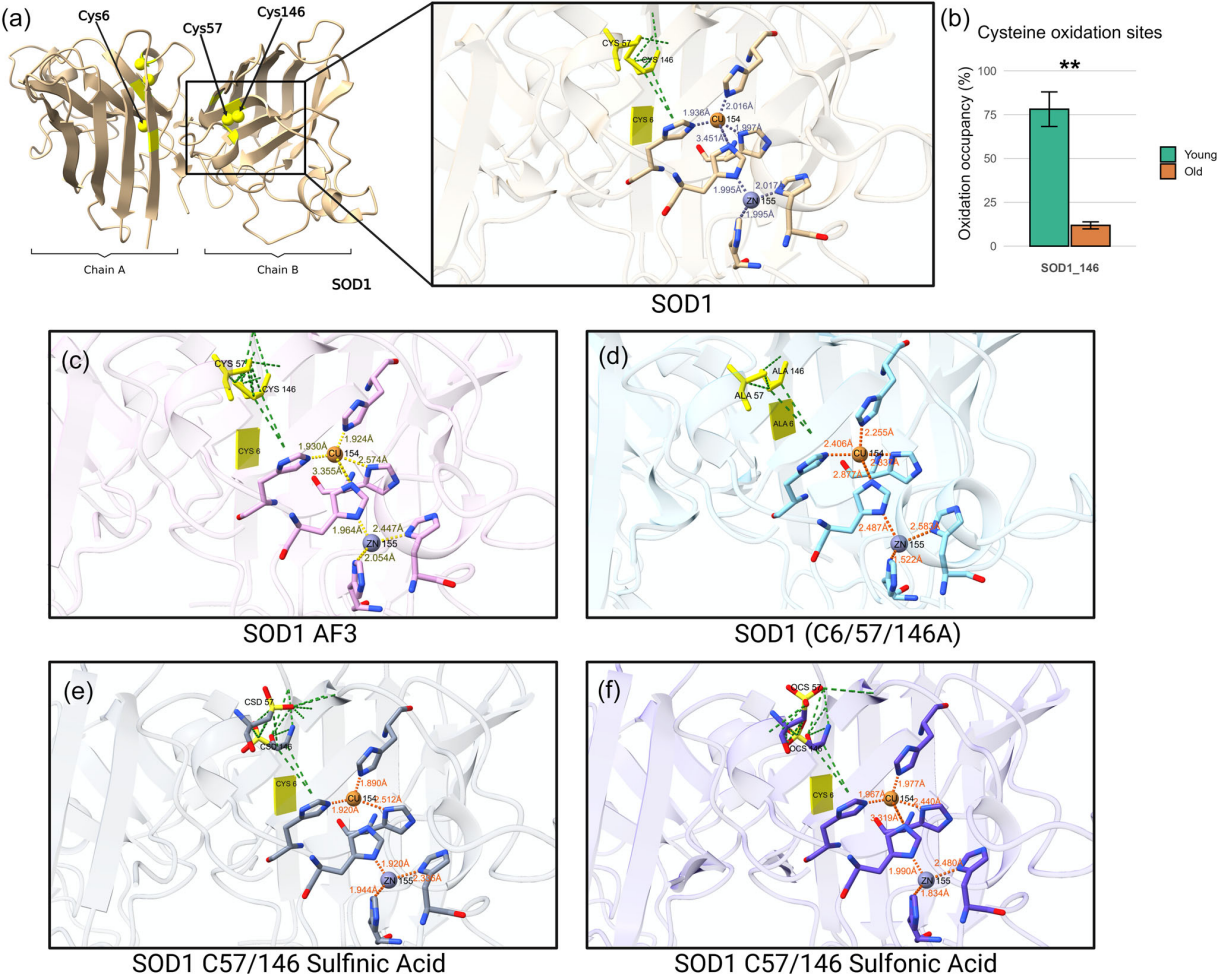
Structural context and redox‐dependent remodelling of SOD1 cysteine residues. (a) Overall structure of the SOD1 homodimer highlighting cysteine residues Cys6, Cys57 and Cys146 (yellow). The boxed region indicates the Cu/Zn metal‐binding active site shown in detail. (b) Quantification of cysteine oxidation occupancy at SOD1 Cys146 in young versus old skeletal muscle. Statistical significance was assessed using pseudo *t*‐tests based on group means, SEs and sample sizes, followed by Benjamini–Hochberg FDR correction. Data are presented as means ± SEM. Significance levels: **P < 0.01. (c) AlphaFold3 model of WT SOD1. (d) AlphaFold3 structural model of the SOD1 (C6/57/146A) mutant, demonstrating disruption of cysteine‐mediated interactions and altered geometry around the metal‐binding site. (e, f) Model of SOD1 with Cys57 and Cys146 oxidised to sulfinic acid (e) and sulfonic acid (f).

Mechanistically, SOD1 harbours a critical disulfide bond between Cys57 and Cys146, which stabilises the β‐barrel architecture and supports the structural environment required for efficient Cu/Zn‐dependent catalysis (Figure 4a) (Arnesano et al., 2004; Baek et al., 2025; Doucette et al., 2004; Lindberg et al., 2004). In skeletal muscle, we observed reduced oxidation at Cys146 in skeletal muscle with ageing (Figure 4b); this is expected to favour a less oxidised thiol state at this position and thereby potentially disfavours Cys57–Cys146 disulfide formation. These data suggest that age‐related remodelling of cysteine redox status occurs at a site directly implicated in SOD1 catalytic competence (Figure 4a,b).

**FIGURE 5:**
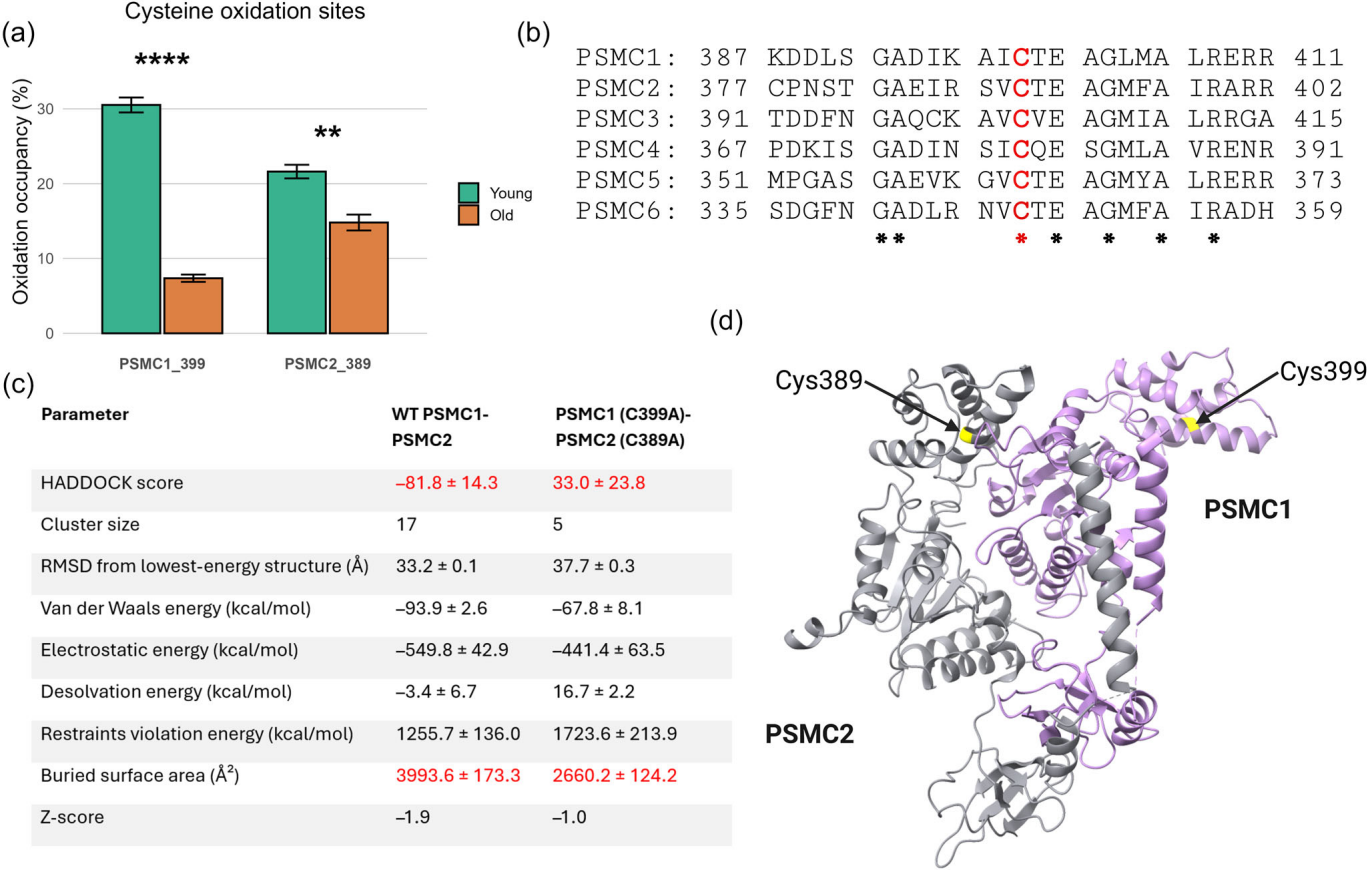
Conserved C‐terminal cysteines stabilise the PSMC1–PSMC2 interface in the 19S ATPase ring. (a) Quantification of cysteine oxidation occupancy at PSMC1 Cys399 and PSMC2 Cys389 in young versus old skeletal muscle. Statistical significance was assessed using pseudo *t*‐tests based on group means, SEMs and sample sizes, followed by Benjamini–Hochberg FDR correction. Data are presented as means ± SEM. Significance levels: **P < 0.01, ****P < 0.0001. (b) Multiple sequence alignment of the C‐terminal conserved segment of mouse PSMCs. (c) HADDOCK docking summary for the PSMC1–PSMC2 interface comparing WT with the Cys‐to‐Ala double mutant PSMC1 (C399A) and PSMC2 (C389A). The HADDOCK score is a weighted sum of intermolecular energy terms, with more negative values suggesting stronger interactions. Compared to WT, the PSMC mutants exhibited a less favourable HADDOCK score, reduced cluster size and buried surface area, and higher restraint violations, indicating a poorly converged and destabilised interface. (d) Structural view of PSMC1–PSMC2 within the 19S ATPase ring of the 26S proteasome, showing the spatial proximity of PSMC1 and PSMC2 at the subunit interface.

To explore the structural consequences of disrupting cysteine redox functionality, we utilised AlphaFold3‐based modelling to generate a non‐redox‐active ‘silent’ mutant in which cysteines were mutated to alanine C6A/C57A/C146A. WT and mutant SOD1 (C6A/C57A/C146A) models maintained high confidence metrics (chain pair interface predicted template modelling (ipTM) > 0.93; average predicted local distance difference test (pLDDT) > 93; ranking score > 0.95) (Figure 4c,d). In SOD1 (C6A/C57A/C146A), the relative positioning and the overall fold of key histidines involved in metal coordination were broadly preserved; however, local changes were observed in the disulfide region and surrounding metal‐site microenvironment, including subtle perturbations in Cu/Zn spacing and ligand geometry compared with the reference model (Figure 4d). These predicted alterations are in line with the loss of the Cys57‐Cys146 disulfide constraint, which has been linked to reduced metal affinity and impaired SOD1 activity.

Finally, we modelled sulfinic and sulfonic states at Cys57 and Cys146 to assess whether over‐oxidation could plausibly disturb the same structural network. Both models maintained high confidence metrics (chain pair ipTM > 0.91; average pLDDT > 91; ranking score > 0.93). Across these models, the overall SOD1 fold and local backbone geometry were largely conserved relative to the reference structure (Figure 4e,f). The principal differences were confined to the modified side chains, where additional oxygen atoms and a higher polarity/negative charge potential were introduced at Cys57/Cys146 without obvious rearrangement of the surrounding metal‐binding architecture (Figure 4e,f). Thus, within the limits of static structure modelling, higher‐order cysteine oxidation at these positions is predicted to be accommodated without major conformational change.

#### 3.3.2 Conserved C‐terminal cysteines stabilise the PSMC1–PSMC2 interface in the 19S ATPase ring

Guided by our PPI network analysis and pathway enrichment, which indicated disrupted redox signalling across multiple core and regulatory proteasome subunits, we used structural modelling to explore potential functional consequences at the level of subunit interactions. The 26S proteasome regulatory subunits, PSMC1 and PSMC2, are AAA+ ATPase components of the 19S regulatory particle and are essential for proteasome function. In skeletal muscle from aged mice, we observed reduced cysteine oxidation at Cys399 in PSMC1 and Cys389 in PSMC2 (Figure 5a). Structural observation revealed that these residues are surface‐exposed and positioned within the C‐terminal region of PSMC proteins, in proximity to the AAA+ ATPase domain, suggesting potential relevance to local structural or regulatory features (Figure 5d). Notably, sequence comparison revealed that this C‐terminal cysteine position is conserved across the mouse PSMC family, supporting the notion that this site represents a conserved feature of proteasomal ATPases (Figure 5b).

**FIGURE 6:**
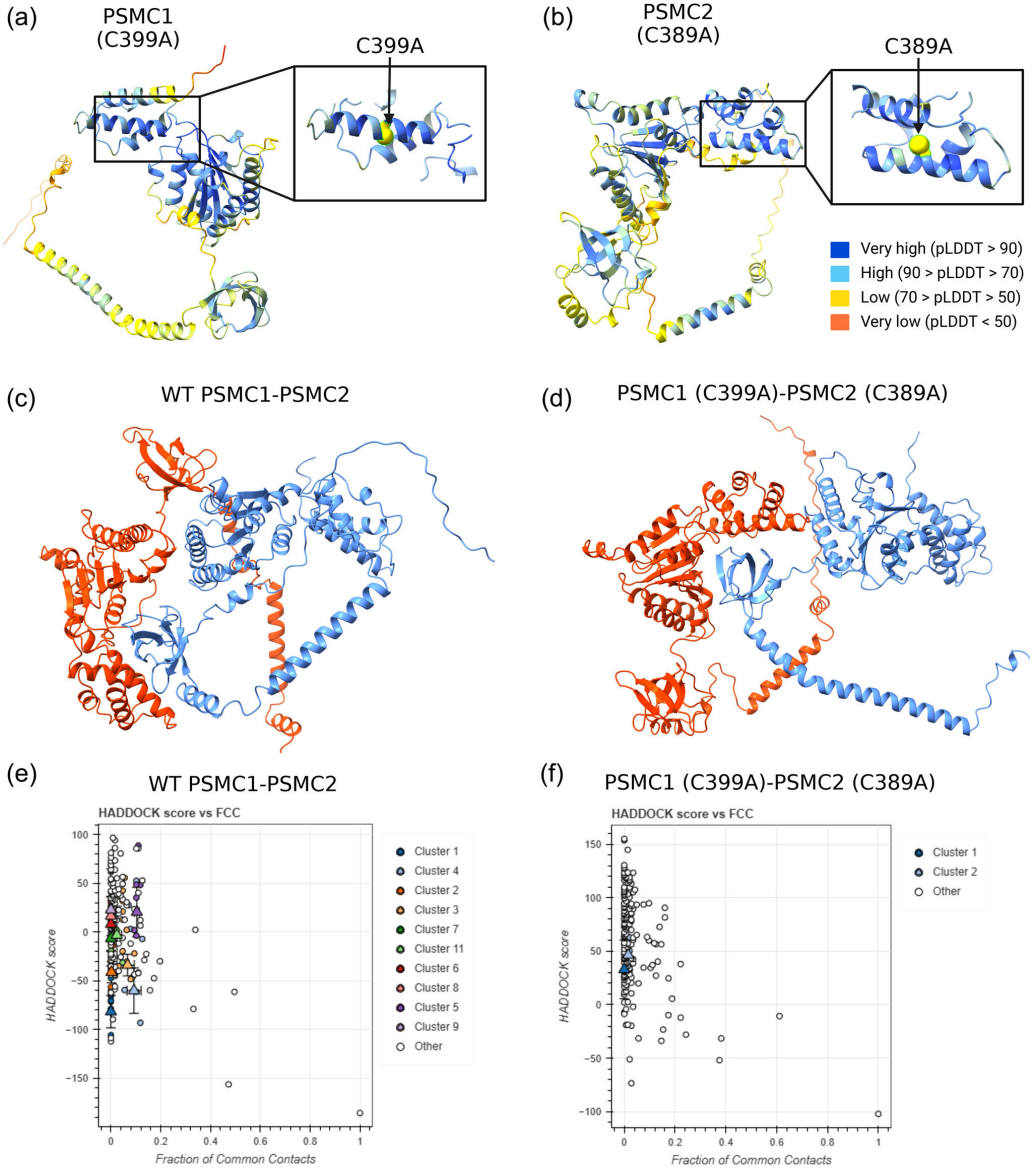
AlphaFold3 modelling of mutant PSMC1 (C399A) and PSMC2 (C389A), and structure‐guided dockings of WT and mutant PSMC1–PSMC2. (a, b) AlphaFold3 structural models of non‐redox‐active PSMC1 (C399A) and PSMC2 (C389A), coloured by per‐residue pLDDT confidence. (c, d) Representative structural views of WT and mutant PSMC1 (C399A)–PSMC2 (C389A) docking simulations. (e, f) WT and mutant PSMC1–PSMC2 docking solutions plotted as HADDOCK score versus fraction of common contacts (FCC), coloured by cluster; more negative HADDOCK scores indicate more favourable predicted interactions.

We therefore hypothesised that these conserved cysteine sites may contribute to PSMC protein function. Non‐redox‐active PSMC1 (C399A) and PSMC2 (C389A) mutants were generated using AlphaFold3 (Abramson et al., 2024). Both models exhibited high to very high pLDDT scores across the C‐terminal region and AAA+ ATPase domain, with AlphaFold ranking scores exceeding 0.73 for both proteins (Figure 6a,b), indicating high confidence in the predicted structures. To define the direct interaction between PSMC1 and PSMC2, we extracted the two subunits from a 26S proteasome structural model and identified inter‐chain atomic contacts using UCSF ChimeraX (Huang et al., 2016; Pettersen et al., 2021). Docking simulations of WT and mutant PSMC1–PSMC2 complexes were then performed using HADDOCK (Figure 6c,d), with active residues selected based on the inter‐chain contact analysis (Honorato et al., 2024). WT PSMC1–PSMC2 docking formed a stable and well‐defined interaction, characterised by a large buried surface area (∼4000 Å^2^), indicating an extensive protein–protein interface, together with favourable van der Waals and electrostatic energy contributions (more negative energy values indicate stronger interactions). In addition, WT docking solutions clustered tightly, reflecting high convergence and reproducibility across independent docking runs (Figure 5c). In contrast, mutation of Cys399 in PSMC1 and Cys389 in PSMC2 to alanine considerably weakened the predicted protein–protein interaction. Mutant complexes demonstrated a lower interface area, unfavourable desolvation energy and higher restraint violations (Figure 5c). Consistent with this, the fraction of common contacts (FCC) analysis demonstrated high convergence among WT docking simulations, whereas the PSMC1 (C399A) and PSMC2 (C389A) mutants docking exhibited low FCC values across clusters, indicating reduced interface similarity and unstable docking solutions (Figure 6e,f). Together, these results suggest that these conserved cysteine residues play a critical structural role in stabilising the PSMC1–PSMC2 interface.

#### 3.3.3 A buried cysteine in MYL6 modulates the energetic landscape of MYL6–MYO18A docking without abolishing binding

**FIGURE 7:**
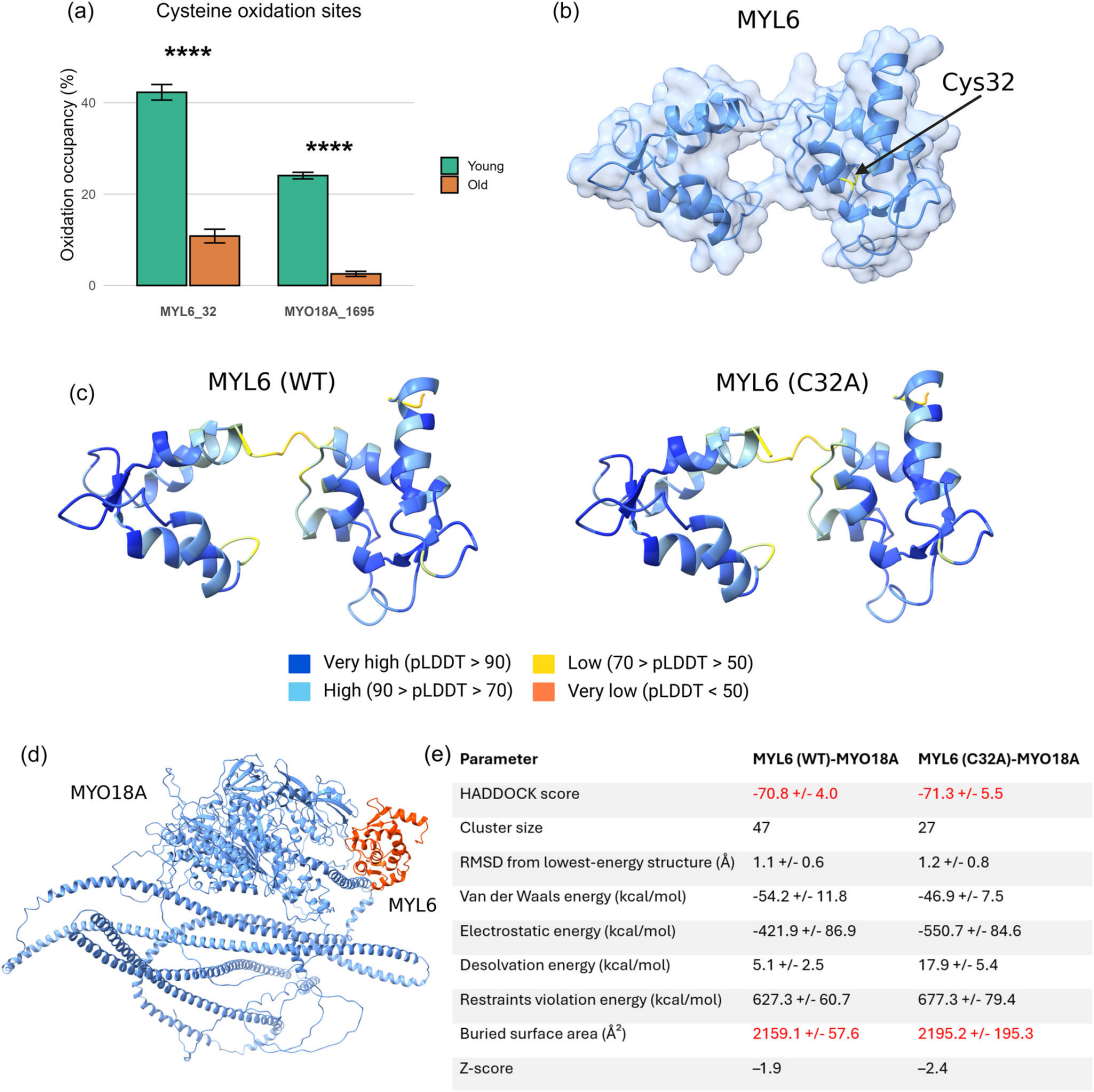
A buried cysteine in MYL6 modulates the energetic landscape of MYL6–MYO18A docking without abolishing binding. (a) Site‐specific cysteine oxidation occupancy at MYL6 Cys32 and MYO18A Cys1695 in young versus old skeletal muscle. Statistical significance was assessed using pseudo *t*‐tests based on group means, SEMs and sample sizes, followed by Benjamini–Hochberg FDR correction. Data are presented as means ± SEM. Significance levels: ****P < 0.0001. (b) Structure of the MYL6 highlighting the buried redox‐sensitive Cys32 residue. (c) AlphaFold3 structural models of MYL6 (WT) and non‐redox‐active MYL6 (C32A), coloured by per‐residue pLDDT confidence. (d) Structural view of predicted MYL6–MYO18A neck‐region complex. (e) HADDOCK docking summary comparing MYL6 (WT)–MYO18A with mutant MYL6 (C32A)–MYO18A. The results were comparable between WT and mutant complexes, indicating that mutation of Cys32 does not substantially alter MYL6–MYO18A binding.

MYL6 is an essential light chain of the myosin complex (Xu et al., 2024) and is predicted to enable microfilament motor activity and participate in muscle filament sliding (KEGG: hsa04814). In our redox proteomics analysis, we observed a significant age‐related reduction in the oxidation of MYL6 at Cys32 (Figure 7a), which is buried within the protein core (Figure 7b). To explore potential structural consequences of this modification, we generated AlphaFold3‐predicted structural models of MYL6 (WT) and a non‐oxidisable MYL6 (C32A) variant; both models displayed high pLDDT values (Figure 7c).

Our results suggest that MYL6 and MYO18A harbour differentially oxidised cysteine residues and are predicted to co‐occur within the same protein–protein interaction network (Figure 3f). Myosin light chains are typically recruited via an IQ (or IQ‐like) helix within myosin heavy chains, which binds calmodulin‐like proteins through a conserved hydrophobic cleft flanked by charged residues (Houdusse et al., 2006; Terrak et al., 2005). Based on this canonical interaction mode, MYL6 is likely to engage the MYO18A IQ helix located in the neck region through complementary hydrophobic packing and electrostatic interactions. We therefore performed HADDOCK docking simulations using these residues as active sites to assess whether perturbation of MYL6 at Cys32, modelled by the C32A variant, alters the predicted MYL6–MYO18A interaction.

HADDOCK docking revealed well‐defined interaction clusters for both WT and C32A MYL6–MYO18A complexes, with comparable HADDOCK scores and low RMSD values, indicating stable complex formation in both conditions. While the overall interface size was preserved between WT and mutant complexes, the energetic composition of the interaction differed. The C32A variant exhibited reduced van der Waals contributions and a higher desolvation penalty, accompanied by enhanced electrostatic interactions and a more favourable Z‐score (Figure 7d,e). Collectively, these results indicate that mutation of the buried Cys32 residue does not abrogate MYL6–MYO18A binding but instead subtly reshapes the energetic landscape of the interaction.

## 4 DISCUSSION

Despite long‐standing interest in ROS as causal drivers of ageing, attempts to extend lifespan in model organisms by boosting antioxidant capacity, particularly via overexpression of antioxidant enzymes, have generally produced limited or inconsistent benefits (Doonan et al., 2008; Gems & Doonan, 2009; Orr et al., 2003; Pérez et al., 2009). This disconnect suggests that ageing is unlikely to reflect a simple, uniform accumulation of oxidative damage. In line with this view, our findings indicated that ageing remodels the redox landscape through selective oxidation of discrete cysteine residues, often in a site‐specific manner, even within the same protein in skeletal muscle. This supports a model in which ageing drives pathway‐targeted modulation of protein function rather than a uniform, global oxidative shift. Importantly, we showed that age‐related cysteine oxidation was not randomly distributed but instead targeted interconnected protein networks involved in mitochondrial metabolic pathways, muscle structure and contraction, and proteostasis, pointing to coordinated remodelling in redox signalling as a hallmark of skeletal muscle ageing.

Our analyses suggested that the proximal amino acid environment could shape cysteine oxidation susceptibility in skeletal muscle, with only modest age‐related differences. We demonstrated that cysteines exhibiting >20% oxidation are flanked by a biased local sequence context characterised by enrichment of basic residues, most prominently arginine and depletion of acidic residues (aspartate and glutamate), with subtle age‐specific differences. This observation is consistent with the original *OxiMouse* motif analysis, which proposed that cysteine oxidation was governed by local sequence and structural features that influence thiol reactivity, electrostatics and disulfide potential (Xiao et al., 2020). Nearby residues modulate the thiol–thiolate equilibrium at physiological pH, with the thiolate being more redox‐reactive. Positively charged residues (e.g., arginine) stabilise the thiolate, while negatively charged residues (e.g., aspartate, glutamate) favour the protonated thiol (Paulsen & Carroll, 2013). However, sequence‐based motif enrichment should be interpreted cautiously: tissue‐to‐tissue differences at the same cysteine residues were reported (Xiao et al., 2020), highlighting that linear motifs alone provide an incomplete and potentially biased view of redox regulation. Instead, residues distant in primary sequence may be proximal in 3D space, shaping electrostatics, solvent exposure and cysteine pKa, while tissue‐specific antioxidant systems and redox dynamics further influence oxidation occupancy (Caporossi et al., 2026; Sies et al., 2022). Thus, the regulation of redox signalling likely reflects an integration of sequence, structure and tissue environment, with ageing altering these interacting constraints in skeletal muscle.

The physiological relevance of selective cysteine oxidation becomes particularly apparent when considering the functions of the proteins affected. Rather than occurring only in isolated biochemical pathways, age‐related redox differences were observed in proteins located at the core of skeletal muscle homeostasis, including those involved in mitochondrial function, muscle contraction and proteostasis (Figure 3). These systems are essential for ATP production, force generation, structural integrity, protein quality control and adaptive remodelling, all of which are progressively compromised during skeletal muscle ageing (Boengler et al., 2017; Ersoy et al., 2024; Hipp et al., 2019; Peterson et al., 2012). Therefore, the observed cysteine oxidation patterns may provide a mechanistic link between altered redox signalling and declining muscle quality, while also highlighting redox‐sensitive nodes that may be relevant to sarcopenia. This is exemplified by CTSB, a lysosomal cysteine protease whose dysregulated activity has been linked to muscle wasting and inflammation, both central features of sarcopenia (Hou et al., 2025; Moon et al., 2016). We observed that CTSB undergoes mixed, site‐specific oxidation remodelling with age, most strikingly a reduction in oxidation at the surface‐exposed Cys211 (Figure 1f). The role of redox signalling is well established in modulating the activity of cysteine cathepsins (Lalmanach et al., 2020; Nagakannan & Eftekharpour, 2017). Therefore, this selective redox remodelling highlights that cysteine‐based signalling nodes could be potential targets for interventions aimed at preserving muscle function during ageing.

To connect the age‐related signatures to mechanisms, we used AlphaFold3 to generate structural predictions for progressive cysteine oxidation states (sulfenic/sulfinic/sulfonic) and non‐redox‐active mutants, prioritising residues likely to act as oxidation‐sensitive mechanistic nodes. This strategy was applied to prioritise functionally important cysteines identified in the dataset, exemplified by the predicted destabilisation of SOD1 metal coordination and dimer stability when the disulfide‐bond‐forming cysteines, Cys57 and Cys146, were mutated to non‐redox‐active alanine.

A conserved C‐terminal cysteine in the PSMC family was uncovered as a potential redox‐sensitive molecular lever controlling PSMC1–PSMC2 association. In silico mutagenesis indicated that substitution of alanine at these cysteine sites (PSMC1 C399A and PSMC1 C389A) destabilises the PSMC1–PSMC2 binding interface, suggesting a direct structural role for these sites in maintaining regulatory particle assembly. Redox‐dependent modulation of 26S proteasome function has been reported previously in skeletal muscle (Fernando et al., 2019). For example, exposure of C2C12 myotubes to H_2_O_2_ increases the expression of muscle‐specific E3 ligases within the ubiquitin–proteasome system, and dysregulated redox signalling has been linked to augmented proteasome‐dependent muscle protein breakdown in vivo (Li et al., 2005; McClung et al., 2009; Powers et al., 2016). However, to our knowledge, this is the first report implicating a cysteine‐dependent redox mechanism within the core AAA+ ATPase machinery of the 26S proteasome. Strikingly, an age‐related decline in oxidation at these cysteine sites was detected in both PSMC1 and PSMC2, implying that redox remodelling during ageing may weaken ATPase subunit interactions and thereby impair proteasomal activity. Future biochemical and structural analyses are needed to directly investigate how redox modification of these cysteines affects 26S proteasome assembly, stability and catalytic activity.

*OxiMouse* proteomics, conceptually inspired by phosphoproteomics, has been a major advance for pinpointing regulatory cysteine residues and linking oxidative post‐translational modifications to protein function (Xiao et al., 2020). However, it is important to recognise that many widely used redox proteomic workflows are oxoform‑selective. Most notably, workflows enriching cysteine sulfenic acids capture transient intermediates that rapidly interconvert into other reversible states (e.g., disulfides or *S*‑glutathionylation). Consequently, such datasets can represent a largely static snapshot of a fundamentally dynamic, non‑linear, multi‑state and emergent redox system. This is especially relevant for proteins containing multiple reactive cysteines where coordinated or competing modifications shape function (Cobley et al., 2025; Li et al., 2025). At the same time, the stoichiometric nature of the *OxiMouse* approach, which reports site‐specific redox occupancy as the fraction of reversibly oxidised relative to total cysteine at each site, offers a quantitative foundation for advancing beyond purely static analyses and toward inference of dynamic redox regulation. In principle, if occupancy measurements were obtained across perturbation and recovery time courses, a Bayesian framework could be applied to infer redox switch kinetics. Conceptually, this would parallel SLAMseq/GRANDSLAM method, which infers RNA turnover dynamics from ratio‐based measurements (Jürges et al., 2018; Rummel et al., 2023). This integration could enable quantitative inference of site‑specific redox dynamics, capturing the non‑linear and context‑dependent nature of redox regulation.

### 4.1 Conclusion

In conclusion, the data presented identify cysteine redox remodelling as a mechanistically informative and physiologically relevant hallmark of skeletal muscle ageing. Rather than reflecting a global oxidative shift, ageing appears to preferentially reconfigure discrete redox‐sensitive nodes embedded within mitochondrial metabolism, muscle contraction and proteostasis. This highlights a potential mechanism through which altered redox signalling may affect physiological processes that maintain skeletal muscle function during ageing. By integrating site‐resolved redox proteomics with structure‐based simulations, this approach enables us to move beyond cataloguing oxidised residues to generate testable mechanistic hypotheses, including a candidate redox‐sensitive cysteine within the core AAA+ ATPase machinery of the 26S proteasome. Importantly, this integration provides a potential translational route: coupling redox proteomics with AI‐enabled structural simulation can prioritise oxidation‐sensitive cysteines as mechanistic nodes and candidate intervention targets for sarcopenia. Finally, a key methodological opportunity is highlighted, moving beyond snapshot redox proteomics toward time‐resolved, quantitative inference of redox kinetics, enabling rigorous tests of whether cysteine switches act as causal drivers of functional change across biological contexts.

## AUTHOR CONTRIBUTIONS

Ufuk Ersoy and Malcolm J. Jackson conceived and designed the work, analysed the data and drafted the manuscript. Both authors critically revised the manuscript. Both authors have read and approved the final version of this manuscript and agree to be accountable for all aspects of the work in ensuring that questions related to the accuracy or integrity of any part of the work are appropriately investigated and resolved. All persons designated as authors qualify for authorship, and all those who qualify for authorship are listed.

## CONFLICT OF INTEREST

None declared.

## FUNDING INFORMATION

None.

## GENERATIVE AI STATEMENT

AI‐based computational tools were used as part of the methodological framework of this study. Specifically, AlphaFold3 was used to generate structural predictions of selected proteins and cysteine oxidation states, and HADDOCK was used for protein‐protein docking simulations, as described in the Methods. In addition, generative AI tools were used only to support language refinement, clarity, and readability during manuscript preparation.

## Supporting information



Tables S1–S4.

## Data Availability

The OxiMouse mass spectrometry proteomics is available online https://oximouse.hms.harvard.edu/.
